# Differential susceptibility effects of the *5-HTTLPR* and *MAOA* genotypes on decision making under risk in the Iowa gambling task

**DOI:** 10.3389/fpsyt.2025.1456490

**Published:** 2025-02-19

**Authors:** Mattias Rehn, Kent W. Nilsson, Cathrine Hultman, Sofia Vadlin, Cecilia Åslund

**Affiliations:** ^1^ Centre for Clinical Research, Västmanland Hospital Västerås, Region Västmanland, Uppsala University, Västerås, Sweden; ^2^ Child and Adolescent Psychiatry, Department of Medical Sciences, Uppsala University, Uppsala, Sweden; ^3^ Division of Public Health Sciences, School of Health, Care and Social Welfare, Mälardalen University, Västerås, Sweden; ^4^ Child Health and Parenting (CHAP), Department of Public Health and Caring Sciences, Uppsala University, Uppsala, Sweden

**Keywords:** serotonin transporter gene, monoamine oxidase A, decision-making, gene-environment interaction, genetic susceptibility, risk-taking, Iowa gambling task

## Abstract

**Introduction:**

The interplay between genetic and environmental factors, as explored through studies of gene-environment interactions (cG×E), has illuminated the complex dynamics influencing behavior and cognition, including decision-making processes. In this study, we investigated the differential susceptibility effects of the *5-HTTLPR* and *MAOA* genotypes on decision-making under risk using the Iowa Gambling Task.

**Methods:**

Data from 264 participants (138 women, 126 men) aged 18-22 years, from the 2015 wave of the Survey of Adolescent Life in Västmanland (SALVe Cohort) was analyzed. Participants provided genetic data including the *MAOA* and *5-HTTLPR* genotypes, and completed the Iowa Gambling Task (IGT) to evaluate decision-making behavior. Parent reports, including assessments of positive parenting styles and early life stress were used for cG×E analysis.

**Results:**

In a General Linear Model, significant interactions were found among males for the *5-HTTLPR*, with SS/SL carriers showing higher net scores with positive parenting and lower scores with less positive parenting in relation to decision-making under risk in the IGT (trials 61-100), indicating differential susceptibility effects. Male LL carriers showed minimal fluctuation in IGT scores. Similar effects were observed for males with the *MAOA* S-allele. No significant interactions were found for females.

**Discussion:**

In conclusion, our study indicates that the *5-HTTLPR* and *MAOA* genes demonstrate susceptibility to environmental factors in influencing decision-making under risk among males, as assessed by the Iowa Gambling Task. We anticipate that these findings will contribute to advancing the understanding of the complex interactions between genetic and environmental factors in shaping human behavior and decision-making.

## 1 Introduction

Decision-making under risk represents a complex cognitive phenomenon shaped by an intricate interplay of genetic, neurobiological, and environmental factors. Understanding the molecular underpinnings of decision-making processes is essential for elucidating individual differences in risk-taking behavior, which holds significant implications across diverse fields such as psychology, neuroscience, and genetics. The Iowa Gambling Task (IGT) is a widely utilized paradigm designed to simulate real-life decision scenarios wherein individuals must navigate trade-offs between short-term gains and long-term losses (1). The present study will investigate how decision-making under risk in the IGT is related to genetic markers of the serotonergic system and their possible interactions with positive and negative environmental factors.

The serotonergic system plays a crucial role in a variety of behavioral and neuroendocrine functions, including the sleep-wake cycle, appetite, aggression, sexual behavior, but also cognition, learning and decision-making, with evidence from both genetic and neurobiological studies (2–4). Serotonin has been shown to modulate decision-making in both rat and human models (4, 5), emphasizing the role of serotonin in shaping decision outcomes.

Two genetic markers that have gathered attention in the research field of decision-making and risk-taking in relation to serotonergic functions are the serotonin transporter gene-linked polymorphic region (*5-HTTLPR*) and monoamine oxidase A (*MAOA*). The serotonin transporter gene (*SLC6A4*) encodes the serotonin transporter protein, which regulates the reuptake of serotonin (5-HT) from the synaptic cleft, thereby modulating serotonin neurotransmission (3, 6). The *5-HTTLPR* polymorphism is a functional variation in the promoter region of the *SLC6A4* gene, resulting in long (L) and short (S) alleles. The presence of the S allele is associated with lower transcriptional efficiency and reduced serotonin reuptake compared to the L allele (6, 7). The S variant of the *5-HTTLPR* has been associated with various conditions and personality traits, including susceptibility to neuroticism and depression, but also pathological gambling (8), risk aversion (9), and response inhibition (10). MAOA is an enzyme involved in the degradation of monoamine neurotransmitters, including serotonin, dopamine, and norepinephrine. The *MAOA* gene contains a polymorphic region, known as the *MAOA-uVNTR* (variable number tandem repeat) where high and low expressing variants influence MAOA enzyme activity and plays a critical role in regulating neurotransmitter availability and signaling (11–13). The variants of the *MAOA-uVNTR* polymorphism are associated with differences in enzyme expression levels and have been linked to various neuropsychiatric conditions and behavioral traits, such as antisocial behaviour and impulsivity (14).

Several studies have investigated the influence of the *5-HTTLPR* on decision-making and risk-seeking behavior. Neukam et al. (15) investigated performance on probability discounting tasks for gains and losses, and found increased risk-seeking for losses in S-carriers of the *5-HTTLPR* independent of acute 5-HT levels. These findings implicate that this genetic variation may confer differences in the early structural development of the neural 5HT-system, possibly influencing risk-taking behaviour. A study investigating the effects of the *5-HTTLPR* on risk perception showed that S-carriers are primarily concerned with the magnitude of outcomes rather than the uncertainty, suggesting a role for this genetic variation in shaping risk perception (16).

Concerning the IGT, the *5-HTTLPR* has been associated with variations in decision-making under ambiguity and risk. He et al. (17) demonstrated that the *5-HTTLPR* genotype influences decision-making in the IGT in a large Chinese sample, with individuals carrying the short (S) allele exhibiting altered decision preferences. Specifically, S allele carriers showed increased risk-taking behavior and impaired decision-making under ambiguity. In a study by Homberg et al. (5) female S allele carriers chose more disadvantageously in trials 41-100 in the IGT compared to L allele carriers. On the other hand, Stoltenberg and Vandever showed that male carriers of the S allele made more advantageous choices than L/L homozygous men on the first block of the IGT (18). Miu et al. (19) further demonstrated that somatic markers of skin conductance responses anticipating the IGT card selection mediate the effect of *5-HTTLPR* on IGT performance, where carriers of the S allele had higher total IGT scores, but almost half of the effect was explained by somatic markers during the IGT. The *5-HTTLPR* has also been investigated in relation to the framing effect, i.e. the tendency to choose risk-aversive when options are presented in the terms of gains, and choose toward gambling and risk-taking when a similar option is presented in terms of losses. Roiser et al. (20) found that S-carriers of the *5-HTTLPR* were more susceptible to framing, which was further investigated by functional magnetic resonance imaging, where S-carriers showed greater amygdala activity when making choices in accord with the frame effect. Gao et al. (21) investigated several potential susceptibility genes of serotonergic and dopaminergic pathways, confirming a higher framing effect in S-carriers of the *5-HTTLPR*, but no effect in relation to the *MAOA* genotype. The *MAOA* has furthermore been investigated in relation to financial risk-taking behavior in several studies yielding mixed findings, further complicated by the diverse range of risk-taking measurements used across studies (22). For instance, Frydman et al. (23) found that male carriers of the *MAOA*-L exhibited higher financial risk-taking behavior compared to those with the high-activity variant, but only in scenarios involving advantageous gambles. However, through a computational choice model, the study revealed that this increased risk-taking behavior was not due to impulsivity but rather the *MAOA*-L carriers’ ability to make better financial decisions under risk. In contrast, Zhong et al. (24) reported that *MAOA*-H individuals displayed higher risk-taking behavior, as shown by a greater preference for longshot lotteries and a lower inclination towards insurance purchases compared to *MAOA*-L individuals. To the best of our knowledge, no previous study has investigated the possible influence of the *MAOA* gene on decision-making and performance in the IGT.

Adverse childhood experiences, including emotional, physical, or sexual abuse, neglect, and household dysfunction, are widely recognized as significant determinants of cognitive and emotional development. Exposure to such early life stressors has been shown to have profound long-term effects on decision-making processes, often due to impairments in cognitive flexibility, which is essential for adapting to changing environments and making sound decisions throughout life (25, 26). Optimal performance on the IGT requires cognitive flexibility (27). However, individuals with a history of adverse childhood experiences often exhibit cognitive perseveration—the opposite of cognitive flexibility—characterized by a tendency to persist with maladaptive strategies despite negative outcomes (25, 26). This inability to adapt is detrimental to performance on the IGT and, by extension, to real-world decision-making, where flexibility is critical for effective problem-solving and risk assessment. Moreover, studies suggest that cognitive perseveration may moderate the relationship between adverse childhood experiences, depression, and other negative outcomes, further complicating the cognitive and emotional landscape for affected individuals (28).

Furthermore, investigations into gene-environment interactions (cG×E) have shed light on the nuanced relationship between genetic factors and environmental influences in relation to behaviour and cognition, including decision-making (14, 29, 30). For example, Chen et al. found interactions between parental warmth and several genetic factors in relation to executive functioning (31). Similarly, Li et al. investigated interactions between parental supervision and the *5-HTTLPR* and found effects in relation to conceptual flexibility (32). A study investigating possible cG×E interactions between the *5-HTTLPR* and childhood trauma in relation to IGT performance found that homozygous carriers of the L allele had lower mean net score under ambiguity (trials 1-20) but not during the later blocks of the IGT (33). Furthermore, childhood trauma was associated with a lower netscore under risk during the later blocks of the IGT. However, no interaction between *5-HTTLPR* and childhood trauma was found in relation to IGT performance (33). A twin study further highlighted the importance of genetic and environmental factors in the etiology of decision making, specifically in relation to IGT performance (34).

In addition to exploring the interaction between genetic factors and decision-making behavior, the hypothesis of “differential susceptibility” to environmental factors holds significant relevance for the research aim. The differential susceptibility theory posits that individuals exhibit varying sensitivity to environmental influences based on genetic variations, suggesting that certain genotypes may confer heightened susceptibility to both positive and negative environmental factors (35–37). In the context of decision-making, the theory of differential susceptibility suggests that genetic factors may moderate the impact of environmental factors on decision-making behavior. For example, individuals carrying certain genetic variants associated with increased serotonin reuptake (e.g., the S allele of *5-HTTLPR*) may exhibit heightened sensitivity to environmental stressors, such as childhood trauma or adverse life events. Consequently, individuals with certain genetic predispositions may exhibit altered decision-making strategies in response to environmental challenges compared to individuals without such genetic predispositions. In contrast, differential susceptibility also convers a heightened sensitivity to positive environmental influences, such as supportive social relationships or positive reinforcement. For instance, individuals with genetic variants associated with higher serotonin transporter efficiency (e.g., the L allele of *5-HTTLPR*) may demonstrate greater responsiveness to positive environmental cues, leading to more adaptive decision-making strategies in rewarding contexts. To further understand the role of environmental factors, this study will examine the influence of both positive parenting and early life stress (ELS) as key determinants of supportive versus negative reinforcing behaviours. Positive parenting represents an important source of environmental support, potentially enhancing adaptive decision-making through positive reinforcement. In contrast ELS is associated with adverse conditions that may amplify vulnerability to negative outcomes. These two factors will provide a comprehensive framework for evaluating how early environmental influences may shape behaviour and decision-making, particularly in individuals with varying genetic susceptibility. The observed differences in previous studies regarding the direction of the main effects of alleles (S vs. L) may thus be better explained by theories of genetic plasticity, i.e., individual differential susceptibility to both positive and negative environmental factors (37, 38). The overall environmental context, encompassing both positive and negative influences on the studied population, may determine which allele is found to be favorable in a given study (14).

Moreover, sex differences in decision-making, as evidenced by studies of the IGT (18, 39, 40) represent an area of ongoing investigation that intersects with the role of early childhood adversity. Research indicates that women and men exhibit different cognitive strategies when engaging with the IGT. Women tend to focus on both win-loss frequencies and long-term pay-off decks, whereas men focus on long-term pay off in the IGT (39). Furthermore, a recent meta-analysis further highlights that males tend to perform better than females on the IGT, potentially linked to differences in certain brain structures related to reward processing and sensitivity to wins and losses (40). Interestingly, a study by Stoltenberg and Vandever found sex by *5-HTTLPR* genotype interactions in relation to IGT performance, but only under ambiguity (trial 1-20) (18). The influence of early life stressors is particularly relevant in understanding these observed sex differences. Females are generally more likely than males to experience adverse childhood experiences (41), a factor known to contribute to a higher prevalence of cognitive perseveration in decision-making tasks (25, 26). This cognitive perseveration can hinder the ability to adapt strategies effectively, underscoring the complex interplay between environmental adversity, genetic predispositions, and sex-specific factors in shaping decision-making patterns. Understanding these dynamics is crucial for developing targeted interventions to support individuals affected by early adversities and improve their decision-making capabilities.

Integrating these concepts into research focused on the Iowa Gambling Task (IGT) is crucial for comprehensively understanding decision-making processes. By elucidating how genetic variations, such as the *5-HTTLPR* and *MAOA* genotypes, potentially interact with environmental factors within the context of the IGT, researchers can gain insights into individual differences in decision-making behavior. Ultimately, this integrated approach, which draws from genetics, neuroscience, and behavioral psychology, can advance our understanding of decision-making and its relevance to neuropsychiatric conditions. It also holds promise for developing personalized interventions aimed at optimizing decision-making abilities and addressing behavioral disorders.

Thereby, the aim of the present study was to investigate differential susceptibility effects of the *5-HTTLPR* and *MAOA* genotypes on decision making under risk in the Iowa Gambling Task. A second aim was to investigate possible sex differences.

## 2 Methods

### 2.1 Study sample

A cohort study (Survey of Adolescent Life in Västmanland, SALVe Cohort) was initiated in 2012 where all adolescents born in 1997 and 1999 in Västmanland, Sweden, were invited to participate along with their guardians. The study was approved by the Ethical Review Board of Uppsala (dnr 2016/569), with an extended approval (dnr 2019-01368). In 2012, adolescents provided written informed consent, completed a questionnaire and provided a saliva sample for genetic analysis. Guardians provided written informed consent and completed a questionnaire. In 2015, wave 2 involved a new collection of questionnaire data from adolescents (n = 1644) and guardians (n = 1607). The present study recruited adolescent participants from the 2015 wave 2 to participate in an experimental session at Västmanland County Hospital in Västerås, Sweden. Participants were invited in a randomized order and consecutively included until the final sample was reached. Criteria for inclusion was available genetic data and no gambling disorder diagnosis. Furthermore, the inclusion procedure accounted for an equal distribution of age and birth year. Upon direct questioning, no participants reported any current or previous history of gambling disorder diagnosis. For a detailed description of the inclusion procedure, see Hultman et al. (42). Data were collected during 2017–2019.

In total, 270 participants aged between 18–22 years were included in the present study. However, a total of 6 participants made repeated selections from one single deck across the entire section of the Iowa Gambling Task, and were excluded from analysis due to insufficient exploration of the reinforcer and punishment contingencies assumed to guide decisions (43). This resulted in a total of 264 participants included in the analyses (138 females, 126 males). In addition, some participants had missing genetic data on the *MAOA*, the *5-HTTLPR*, or the parent reports, resulting in a different n in the respective analytic models, as reported in the Tables. Estimating the sample size required to achieve sufficient statistical power in our study is challenging. Although cG×E interaction analyses typically require large sample sizes, some argue that well-designed experimental studies allow for greater control over variable assessment (44, 45). Therefore, smaller sample sizes may be adequate. Given the exploratory nature of this study, the current sample size was considered appropriate to detect tendencies in differential susceptibility.

Independent sample t-tests were conducted to compare the current study’s subsample (N = 270) with the broader cohort (N = 1215) in terms of self-reported symptoms assessed using the Adult ADHD Self-Report Scale (46), Depression Self-Rating Scale (47), and the Adult Anxiety Scale-15 (48). The results indicated no significant differences in self-reported symptoms of depression (p = 0.961) or ADHD (p = 0.543) between the subsample and the cohort. However, the current subsample reported significantly lower levels of anxiety symptoms compared to the cohort (p = 0.015), as previously reported (49).

### 2.2 Experimental procedure

Upon arrival at the experimental session, detailed information about the procedure was provided by the examiner. Informed consent had been obtained from participants in an earlier part of the study, and specific consent for the experimental sub-study was subsequently sought, ensuring that all participants were aware of and agreed to the specific conditions and procedures involved. Participants completed several tasks during the experimental session, including a battery of questionnaires (on gambling, gaming, personality traits, sleep habits, sensory processing sensitivity, and positive/negative affect), four emotion cognition tasks, two interviews on substance- and behavioral addictions, and three different gambling tasks. Participants were reimbursed a gift card with a fixed amount of 1000 SEK (≈ 100 €) for participation, and were also informed that they would receive additional gratification depending on their performance in the three gambling tasks. The maximum additional amount was 200 SEK/task (≈ 20 €). The IGT considered in the current study was administered as the second out of three gambling tasks during the latter part of the session.

### 2.3 Iowa gambling task

A computerized version of the original Iowa Gambling Task developed by Bechara and colleagues (43) was administered, featuring four virtual card decks: two disadvantageous (A and B) and two advantageous (C and D). Participants repeatedly selected one card at a time from these decks over 100 trials, aiming to maximize their profits. The original task was modified with visual and auditory stimuli to simulate a casino environment. Consistent with the instructions provided in Bechara et al. (50) participants were only informed that some decks were better than others, which has been shown to enhance task performance (51, 52). Each participant began with a starting credit of 2000 SEK. Two progress bars displayed the accumulated earnings and losses at the top of the screen, with the total earnings shown on the right. Participants selected decks using a computer mouse, revealing a card that indicated both a gain and a loss. The net sum of each card was either added to or subtracted from the total earnings. Positive amounts were accompanied by a winning sound, while negative amounts were followed by a losing sound. Participants were free to switch between decks as often as they liked. Consistent selection from decks A or B over 10 trials resulted in a net loss of 250 SEK, whereas selecting from decks C or D over 10 trials resulted in a net gain of 250 SEK.

Net scores were calculated by subtracting the number of selections from the disadvantageous decks from those of the advantageous decks, represented as (C+D)-(A+B). Net scores > 0 indicate a tendency towards the advantageous choices, whereas net scores < 0 indicate a tendency towards the disadvantageous choices. The task was divided into five blocks, each comprising 20 rounds. Net scores specifically focused on the last 40 trials (trials 61–100), which are commonly referred to as decision-making under risk (53). Previous research from our group has demonstrated that stable choice patterns typically emerge during block 4, specifically rounds 61 to 100 (49).

### 2.4 Positive parenting

Assessments of positive parenting style were used to explore the influence of this positive environmental factor on differential susceptibility effects in IGT decision-making. Positive parenting style was assessed using the parent report of the Parents as Social Context Questionnaire (PASCQ) (54), Swedish version (55), during wave 2 when the participants were either 15-16 or 17-18 years old. The parent report of PASCQ was completed by the participating guardian. The questionnaire is a 30-item self-report scale that evaluates six parenting styles across two dimensions: a positive dimension and a negative dimension. The positive dimension includes parenting styles such as warmth (e.g., “I really know how my child feels about things”), structure (e.g., “I make it clear to my child what I expect from him/her”), and autonomy support (e.g., “I encourage my child to express his/her opinions even when I don’t agree with them”). The negative dimension includes parenting styles such as rejection (e.g., “Sometimes my child is hard to like”), chaos (e.g., “When my child gets in trouble, my reaction is not very predictable”), and coercion (e.g., “To get my child to do something, I have to yell at him/her”). Each parenting style is assessed through five questions, with response options ranging from “not at all true” (0) to “very true” (3). A positive summation index, termed PASCQ^POSITIVE^, which includes only the positive parenting styles, was created using the 15 positive items, with a possible score range of 0 to 45 points. The Swedish version of the PASCQ has been psychometrically evaluated and is reported as suitable for measuring the six parental dimensions (α = .617, RMSEA = .054, SRMR = .061). For further details see Keijser et al. (55). Both main and cG×E interaction effects of PASCQ^POSITIVE^ on net scores during trials 61-100 were analyzed.

### 2.5 Early life stress

The influence of a negative environmental factor on differential susceptibility effects in IGT decision-making was explored through assessments of stressful events during childhood. Early life stress (ELS) was assessed through the completion of the Neuropattern Questionnaire–Pre-/postnatal-Stress-Questionnaire (NPQ–PSQ) by guardians during wave 2 (56). The NPQ–PSQ is a retrospective self-report instrument that evaluates ELS across four dimensions: pregnancy (e.g., relationship status), birth (e.g., special medical interventions post-birth), childhood (e.g., significant family conflicts), and general information (e.g., estimated income during childhood). The NPQ–PSQ is part of the NeuroPattern, a translational tool designed to detect and address stress-related pathology. The NPQ–PSQ has demonstrated adequate psychometric properties (56, 57). The NPQ–PSQ was translated into Swedish by researchers in the SALVe cohort group following recommended procedures (58, 59). A summation index was constructed, comprising 19 specific items and one open-ended question assessing the presence (yes/no) of various stressors during participants’ childhood (range 0-20). The mean score of the index was 1.8, and it was dichotomized as follows: scores ranging from 0 to 1 were categorized as below the mean (0), while scores of 2 and above were categorized as above the mean (1). Both main and cG×E interaction effects of ELS on net scores during trials 61-100 were analyzed.

### 2.6 Genotyping

Genomic DNA was extracted from saliva samples using the DNA Self-Collection Kit (Oragene^®^, Ottawa, Canada), following the manufacturer’s guidelines. The 30-bp variable number tandem repeat (VNTR) polymorphism of *MAOA* (*MAOA-uVNTR*) was analyzed as previously described (60). Five variants of the 30-bp repeat sequence were identified: 2, 3, 3.5, 4 and 5. Based on the length, the 2 and 3 repeats were categorized as short (S) and 3.5, 4 and 5 repeats were coded as long (L). Among males, individuals were classified as carriers of the S-allele (S) or carriers of the L-allele (L). Among females, individuals were classified as carriers of the SS or LS genotypes (SS/LS), and as homozygous carriers of the L-allele (LL).

The *5-HTTLPR* polymorphism was analyzed following the methodology described previously (61). For analyses, both *MAOA* and *5-HTTLPR* were categorized into two groups: carriers of the short allele (S, SS and LS), coded as 0, and homozygous carriers of the long allele (L, LL), coded as 1.

For a detailed description of the genetic analysis process, see **Supplementary Material**.

### 2.7 Statistical analysis

The results presented were primarily generated using IBM SPSS Statistics version 28. Significance threshold was set to p < 0.05.

Mann-Whitney U tests or Chi-square (*χ²*) tests were employed to assess differences between sexes in the descriptive section. For genotype analysis, we utilized the R package “HardyWeinberg” to confirm Hardy-Weinberg equilibrium. The *5-HTTLPR* polymorphism was tested using the standard formula with one degree of freedom. The *MAOA* polymorphism was analysed using a method that accounts for the X chromosome, employing two degrees of freedom. This approach was necessary because males have only one X chromosome and therefore do not have the heterozygous genotype typically characterized by Long/Short alleles.

The outcome net score for IGT trials 61-100 was calculated with a total mean score on advantageous decks minus disadvantageous decks [(C+D) - (A+B)] over 40 trials, starting at trial 61. The possible range of scores was -40 to 40. Visual inspection of the histogram suggested that the net scores variable should be treated as a continuous variable, although it did not conform to a normal distribution.

In statistical analyses General Linear Models (GLMs) were conducted, employing the robust HC0 method for heteroskedasticity-consistent standard errors. A robust approach was deemed necessary because the data did not fully meet all the assumptions required for a GLM. Effect sizes were reported as partial eta squared (η_p_^2^) to interpret the magnitude of each covariate’s impact in the models. Effect size estimation followed (62) small=0.01, medium=0.06 and large=0.15.

GLMs with a single covariate model were applied to the predictors: sex, ELS, PASCQ^POSITIVE^, *5-HTTLPR*, and *MAOA* to analyse any differences in outcome net scores. Two cG×E interaction models were tested for sex interaction effects, involving two-, three-, and four-way interactions. Thereafter, further analyses were divided by sex, with separate models for females and males. Three different models were analyzed for each genotype: Model 1 included ELS, Model 2 included PASCQ^POSITIVE^, and Model 3 included both ELS and PASCQ^POSITIVE^.

## 3 Results

**Table 1** provides a demographic description, illustrating the totals and univariate differences between females and males. The distribution of age and sex was expected to be equal across the groups. Regarding ELS, 64.8% of the study population (males: 63.5%, females: 65.9%) experienced at least one early life stress factor, with 25.8% experiencing three or more such factors. A significant difference in the net score of the Iowa Gambling Task (IGT) trials 61-100 was observed between females and males, with males selecting more advantageous decks. Sex differences in learning rate, calculated using net scores for each 20-trial block, indicated similar net scores between males and females during the early trials, with higher net scores emerging in males toward the end of the task (see **Supplementary Figure 1**). No significant differences were found between sex and the covariates. The genotypes were in Hardy-Weinberg equilibrium when tested across the entire group.

**Table 1:** Descriptive characteristics of the study sample.

Variables	Total	Females	Males	Statistics
	(*n*=264)	(*n*=138)	(*n*=126)	
Continuous	*M* (*SD*)	*M* (*SD*)	*M* (*SD*)	Mann-Whitney U test
Age	20.13 (0.800)	20.19 (0.856)	20.06 (0.731)	*z*=-1.041, *p*=0.298
Net score (IGT trials 61-100)	8.27 (16.815)	6.44 (14.537)	10.29 (18.855)	*z*=-2.082, *p*=0.037
PASCQ^POSITIVE^	36.79 (5.106)	37.15 (5.265)	36.40 (4.915)	*z*=-1.162*, p*=0.245
Nominal	*n* (%)	*n* (%)	*n* (%)	HWE test
*5-HTTLPR*				*χ²*(1)=0.006, *p*=0.940
SS	36 (14.17)	15 (11.36)	21 (17.21)	
LS	120 (47.24)	62 (46.97)	58 (47.54)	
LL	98 (38.58)	55 (41.67)	43 (35.25)	
*MAOA*				*χ²*(2)= 3.411, *p*=0.182
S/SS	59 (22.52)	18 (13.24)	41 (32.54)	
LS	72 (27.48)	72 (52.94)		
L/LL	131 (50.00)	46 (33.82)	85 (67.46)	
Dichotomous	*n* (%)	*n* (%)	*n* (%)	Pearson *χ²*
Early life stress (ELS)				*χ²*(1)=0.815, *p*=0.367
Below mean	148 (58.73)	74 (56.06)	74 (61.67)	
Above mean	104 (41.27)	58 (43.94)	46 (38.33)	

### 3.1 Net score (IGT trials 61-100)

A GLM using one predictor at a time identified a significant relationship between net score on IGT trials 61-100 and the *5-HTTLPR* genotype in females (**Table 2**). Female SS/SL carriers exhibited an increase in net score, whereas female LL carriers showed a decrease in net score. Differences in learning rate was also indicated by higher net-scores emerging during the end of the task in female SS/SL carriers (see **Supplementary Figure 2**). There were no significant differences in block-wise net-scores between *MAOA* variants, in males or females (see **Supplementary Figure 3**). No other covariates in the analysis were found to have a statistically significant impact on net score.

**Table 2:** General linear model with a single covariate conducted on the outcome net score (IGT trials 61-100) for the total population, females and males.

Variables	Total				Females				Males			
	*n*	*B* (95% CI)	Effect size (η_p_^2^)	*p*	*n*	*B* (95% CI)	Effect size (η_p_^2^)	*p*	*n*	*B* (95% CI)	Effect size (η_p_^2^)	*p*
Sex (0=Females, 1=Males)	264	3.851 (-0.241; 7.943)	0.013	0.065								
*5-HTTLPR* (0=SS/SL, 1=LL)	254	-2.641 (-6.819; 1.537)	0.006	0.214	132	-4.857 (-9.582; -0.132)	0.031	0.044	122	0.516 (-6.615; 7.648)	0.000	0.886
*MAOA* (0=SS/SL, 1=LL)	262	2.076 (-2.015; 6.167)	0.004	0.319	136	0.122 (-5.200; 5.443)	0.000	0.964	126	1.725 (-5.601; 9.052)	0.002	0.642
ELS (0=Below mean, 1=Above mean)	252	-0.388 (-4.554; 3.777)	0.000	0.854	132	2.164 (-2.654; 6.982)	0.006	0.376	120	-2.863 (-9.964; 4.239)	0.005	0.426
PASCQ^POSITIVE^	253	0.061 (-0.389; 0.511)	0.000	0.790	133	-0.377 (-0.883; 0.129)	0.016	0.143	120	0.676 (-0.128; 1.480)	0.023	0.099

### 3.2 Net score (IGT trials 61-100) in interaction models

Given that several studies have demonstrated sex differences in the context of cG×E involving both *5-HTTLPR* and, specifically, *MAOA*, we initially explored models incorporating main effects, as well as two-, three-, and four-way interaction effects of both candidate genes. These models included the following interactions: Sex × *5-HTTLPR* × ELS × PASCQ^POSITIVE^, and Sex × *MAOA* × ELS × PASCQ^POSITIVE^. Sex did not substantially influence the models. However, to avoid potential biases associated with sex, such as the X-linked nature of the *MAOA* gene—where males have only one allele copy inherited from their mother—we opted to conduct sex-separated final analyses.

A GLM analysis was conducted to test three different interaction model types for each genotype. Model 1 (ELS) and Model 2 (PASCQ^POSITIVE^) consisted of one interaction term, while Model 3 included two interactions terms (ELS and PASCQ^POSITIVE^). The effects of these models were assessed on net score (IGT trials 61-100).

**Figure 1:**
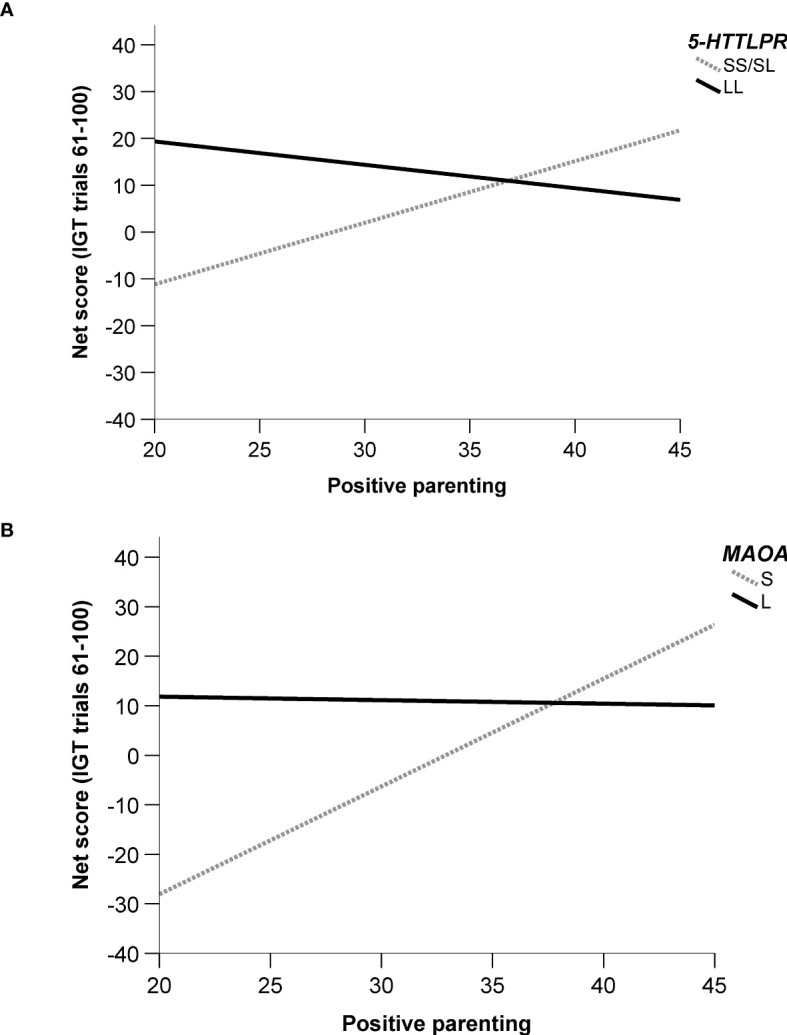
Regression line plot of significant two-way interaction models, showing the relationship between net score (IGT trials 61-100) and positive parenting. **(A)** *5-HTTLPR* x PASCQ^POSITIVE^ – Males. **(B)** *MAOA* x PASCQ^POSITIVE^ – Males.

In males, significant interaction effects were observed between *5-HTTLPR* and PASCQ^POSITIVE^ in Model 2 and Model 3 (see **Table 3**). Male SS/SL carriers showed an increase in net score with greater positive parenting, whereas male LL carriers exhibited a decrease in net score under similar conditions (see **Figure 1A**).

**Table 3:** General linear model with multiple predictors.

		Females				Males			
Model type	Variables	*n*	*B* (95% CI)	Effect size (η_p_^2^)	*p*	*n*	*B* (95% CI)	Effect size (η_p_^2^)	*p*
Model 1	*5-HTTLPR*		-8.146 (-23.496; 7.205)	0.009	0.296		6.959 (-14.770; 28.687)	0.004	0.527
	ELS		1.181 (-5.771; 8.134)	0.001	0.737		-1.505 (-10.251; 7.242)	0.001	0.734
	*5-HTTLPR* x ELS		2.256 (-7.119; 11.632)	0.002	0.635		-4.109 (-19.318; 11.100)	0.003	0.594
		126				116			
Model 2	*5-HTTLPR*		-1.058 (-35.675; 33.558)	0.000	0.952		66.848 (14.101; 119.596)	0.053	0.013
	PASCQ^POSITIVE^		-0.111 (-0.763; 0.541)	0.001	0.737		1.316 (0.424; 2.208)	0.071	0.004
	*5-HTTLPR* x PASCQ^POSITIVE^		-0.107 (-1.002; 0.788)	0.000	0.814		-1.816 (-3.252; -0.379)	0.053	0.014
		127				116			
Model 3	*5-HTTLPR*		-0.601 (-41.525; 40.324)	0.000	0.977		77.598 (19.234; 135.961)	0.060	0.010
	ELS		1.226 (-6.344; 8.795)	0.001	0.749		-0.172 (-8.906; 8.562)	0.000	0.969
	PASCQ^POSITIVE^		0.014 (-0.683; 0.712)	0.000	0.968		1.315 (0.389; 2.240)	0.068	0.006
	*5-HTTLPR* x ELS		2.023 (-7.743; 11.789)	0.001	0.682		-6.307 (-21.454; 8.840)	0.006	0.411
	*5-HTTLPR* x PASCQ^POSITIVE^		-0.189 (-1.100; 0.722)	0.001	0.682		-1.852 (-3.321; -0.383)	0.054	0.014
		126				115			

Analyses of *5-HTTLPR* on outcome net score (IGT trials 61-100) for females and males, employing three different model types (Model 1-3).

Similarly, significant interaction effects were observed among males between the *MAOA* genotype and PASCQ^POSITIVE^ in both Model 2 and Model 3 (see **Table 4**). Male S carriers showed an increase in net score with increasing positive parenting, whereas male L carriers demonstrated a decrease in net score under the same conditions (see **Figure 1B**).

**Table 4:** General linear model with multiple predictors.

		Females				Males			
Model type	Variables	*n*	*B* (95% CI)	Effect size (η_p_^2^)	*p*	*n*	*B* (95% CI)	Effect size (η_p_^2^)	*p*
Model 1	*MAOA*		-7.329 (-23.850; 9.192)	0.006	0.382		-3.846 (-26.483; 18.791)	0.001	0.737
	ELS		0.695 (-5.323; 6.713)	0.000	0.820		-5.307 (-18.037; 7.424)	0.006	0.411
	*MAOA* x ELS		4.468 (-5.921; 14.858)	0.006	0.396		3.631 (-11.681; 18.942)	0.002	0.639
		130				120			
Model 2	*MAOA*		31.876 (-7.950; 71.703)	0.019	0.116		85.115 (29.198; 141.031)	0.073	0.003
	PASCQ^POSITIVE^		-0.110 (-0.702; 0.483)	0.001	0.715		2.181 (1.074; 3.288)	0.116	<0.001
	*MAOA* x PASCQ^POSITIVE^		-0.855 (-1.879; 0.169)	0.021	0.101		-2.254 (-3.748; -0.761)	0.072	0.003
		131				120			
Model 3	*MAOA*		17.601 (-31.284; 66.486)	0.004	0.477		83.968 (20.875; 147.061)	0.058	0.010
	ELS		0.505 (-5.684; 6.694)	0.000	0.872		-2.526 (-13.270; 8.217)	0.002	0.642
	PASCQ^POSITIVE^		-0.102 (-0.710; 0.507)	0.001	0.742		2.150 (1.020; 3.280)	0.112	<0.001
	*MAOA* x ELS		2.413 (-8.370; 13.196)	0.002	0.659		0.521 (-13.290; 14.333)	0.000	0.941
	*MAOA* x PASCQ^POSITIVE^		-0.581 (-1.649; 0.486)	0.009	0.283		-2.240 (-3.762; -0.717)	0.070	0.004
		130				119			

Analyses of *MAOA* on outcome net score (IGT trials 61-100) for females and males, employing three different model types (Models 1-3).

In contrast, no significant interaction effects were found for females for either the *5-HTTLPR* or *MAOA* genotype (**Tables 3**, **4**).

## 4 Discussion

In the present study, we found indications of a gene-environment interaction involving positive parenting with both the *5-HTTLPR* and *MAOA* genotypes, influencing the net score of the Iowa Gambling Task in males. These findings suggest that the *5-HTTLPR* and *MAOA* genotypes exhibit differential susceptibility effects on decision-making under risk in the Iowa Gambling Task.

Previous research has highlighted the “risk” and “sensitivity” properties of the short variants of these genes. For instance, Caspi and colleagues (63) demonstrated that maltreated children with the short variant of *MAOA* were more likely to develop antisocial problems. Similarly, Caspi and colleagues (64) reported that S-carriers of the *5-HTTLPR* displayed elevated depressive symptoms and suicidality following stressful life events and childhood maltreatment.

Among male carriers of the short variants of the *5-HTTLPR* and the short variant of the *MAOA* gene, we observed a significant cG×E interaction with positive parenting. S-carriers with low scores on positive dimensions of parenting styles—warmth, structure, and autonomy support—showed markedly lower IGT net scores, whereas S-carriers with high scores on positive parenting had the highest IGT net scores among all males. In contrast, male carriers of the long variant showed minimal fluctuation in IGT net scores due to parenting style.

The lack of results among females may have several explanations. Males tend to take more risks on the IGT compared to females, reflecting overall risk-taking behavior. Additionally, males exhibit better task performance and greater lateralized brain activity than females (65). Research has shown that females require 40-60 additional trials before they reach the same level of performance as males (39). These performance differences are associated with variations in activity in the orbitofrontal cortex and dorsolateral prefrontal cortex, as well as differences in serotonergic activity and left-right hemispheric activity (39), both of which may be influenced by the *5-HTTLPR* and *MAOA* genes. A more recent meta-analysis (40) of sex differences reached the same conclusion: males obtained higher total net scores than females. The results were not moderated by mean sample age, publication year, sample size, study quality, type of monetary reward (i.e., real or simulated), task version (i.e., computerized or manual), or the geographical region in which the study was conducted (based on continent), nor were they influenced by publication bias. Drawing on previous research that highlights functional sex-related asymmetries in the ventromedial prefrontal cortex and amygdala, as well as differences in sensitivity to wins and losses (40), one might speculate that these IGT-related sex differences are more profound than potential environmental or genetic factors. Consequently, a more nuanced IGT study design may be necessary to investigate cG×E interactions among females. Such a design could incorporate sex-specific environmental influences. For instance, females are more likely than males to encounter negative environmental exposures such as sexual assault and child sexual abuse, whereas males are more likely to experience accidents, non-sexual assaults, and the witnessing of death or injury (66). However, less is known about sex differences in exposure to positive environmental factors, which warrants further exploration.

Given that we employed a 100-trial design, one might speculate that if we had repeated the experiment, females could have demonstrated more stable results, thereby mimicking real-world decision-making. In fact, we have observed more pronounced susceptibility to gene-environment interactions related to both the *5-HTTLPR* and *MAOA* genes among females, as evidenced by our previous findings (67). Therefore, the sex differences observed in the IGT may reflect broader gender differences in the regulation of emotions (14, 39, 68). With a more extensive experimental design, the task might prove equally predictive for females as it is for males.

If we consider the IGT net score as a relatively accurate measure of decision-making processes, it could also serve as a proxy for individual differences in risk-taking behavior. Indeed, our present findings echo results from the initial *MAOA* cG×E interaction study by Caspi and colleagues (63), as well as early work by our group (69, 70). However, real-life decision-making typically involves multiple factors, which have raised questions about the usefulness of the IGT in predicting long-term behavioral trajectories. Concerns about the IGT include the lack of a clear definition of the specific aspects of decision-making it measures, limited data on its reliability, and the potential influence of personality traits and mood states on task performance (71). Moreover, our study demonstrates that both genetic and environmental factors have an impact on IGT net scores, with environmental factors often changing over time, further complicating its long-term predictive capacity.

Moreover, earlier studies of *MAOA* cG×E interactions focused exclusively on negative environments. Since the differential susceptibility theory suggests that individuals exhibit varying susceptibility to both positive and negative environmental factors (35–37), hypothesis-driven statistical modeling is theoretically necessary in studies of cG×E models, where the gene’s effect on the outcome variable depends on other predictor variables (14). In the context of decision-making, the theory of differential susceptibility posits that genetic factors may moderate the impact of environmental factors, or vice versa, on decision-making behavior. Our model supports the assumption that *5-HTTLPR* and *MAOA* exhibit susceptibility properties, consistent with studies on depression, suicide (64), criminality (63, 69), and alcohol problems (72, 73). In our study, these genetic influences were reflected in high IGT net scores as equivalents to low scores on conduct problems, criminality, or negative alcohol consumption. Although, to the best of our knowledge, there is no previous study on gene-environment interaction susceptibility properties in relation to *5-HTTLPR*, *MAOA*, and IGT net score, our study thereby aligns well with a substantial body of knowledge on other outcome variables related to both good and poor decision-making and the *MAOA* gene.

In the context of genetic plasticity (35, 36), the concepts of resilience and vulnerability are often discussed as two opposing yet interconnected outcomes of how individuals respond to environmental challenges. It is important to recognize that protective and risk factors in life are interconnected. As we have previously shown, both the *5-HTTLPR* and *MAOA* genes can promote adaptive responses to stress, with certain polymorphisms interacting differently depending on positive or negative environmental factors, and also differing between males and females (37, 38, 67, 73). Additionally, resilience and vulnerability may be viewed by some researchers as opposing ends of a continuum, while others consider them fundamentally different concepts (74, 75). From a biological perspective, we argue that resilience and vulnerability should not be seen as fixed traits, but rather as continuously ongoing processes that interact with environmental resilience and vulnerability factors in a complex web of life circumstances. As described by Plomin and colleagues, one environmental factor that may account for a small percentage of variance across all individuals can explain nearly all the variance for a specific subgroup (76).

Moreover, we have previously raised the question of whether positive environmental factors (E-pos) should be regarded as general resilience factors that benefit all individuals equally—both those significantly impacted by negative environmental (E-neg) life events and those without such experiences—or if certain E-pos factors are specifically relevant in the context of adversity, potentially operating only in individuals with particular E-neg factors. To our knowledge, these questions have largely been overlooked in the research on cG×E, both from the traditional diathesis-stress perspective and within the framework of differential susceptibility (14). Therefore, we believe that the field of cG×E research has only just begun to explore the nuances of resilience and vulnerability.

In a previously conducted study, we identified two-, three-, and four-way interactions between the *5-HTTLPR*, positive and negative family environments, and sex in relation to both depressive symptoms and delinquency (37). However, the susceptibility properties of *5-HTTLPR* were notably less pronounced in relation to depressive symptoms compared to delinquency. This observation potentially underscores the role of the serotonin transporter gene in decision-making processes and risk-taking behaviors. In the present study, we did not observe any significant sex-by-genotype interactions in our cG×E models. However, it is noteworthy that males exhibited higher IGT net scores, which could potentially obscure any sex interaction effects. A previous study by Stoltenberg and Vandever (18) reported sex-by-*5-HTTLPR* interactions in relation to IGT performance. They found that male carriers of the S-allele made more advantageous choices than L/L carriers during the initial block of the IGT, when participants were navigating the game under conditions of ambiguity.

Since *MAOA* is located on the X-chromosome, a challenge arises in the cG×E approach. Females possess two X chromosomes, whereas males have only one, resulting in heterozygosity present in females but not in males. The expression of *MAOA* in heterozygous allele carriers remains unclear, leading many researchers to exclude heterozygous females from their samples or focus exclusively on males (77). In contrast, heterozygous effects have been analyzed for the serotonin transporter gene *SLC6A4* and the *5-HTTLPR* polymorphism. Given the limited sample size and consistent with prior studies, we opted to combine individuals homozygous for the short alleles (SS) with the heterozygous individuals (SL) (78).

In the present study, we did not observe any effects of early life stress on IGT net scores, either directly or as interaction effects. A previous study investigating cG×E interactions between the *5-HTTLPR* and childhood trauma in relation to IGT performance similarly found no significant effects (33). However, that study reported an association between childhood trauma and lower net scores under risk, which is partly suggested by the borderline significance observed among males in relation to ELS in our current study. In a recent study on the prevalence of adverse childhood experiences in the United States, Giano and colleagues found that 57.8% of participants reported having experienced at least one adverse childhood experience, while 21.5% indicated that they had encountered three or more (41). The prevalence of early life stress in our sample was slightly higher, which may be attributed to a random variation due to a small sample, as well as the use of a different questionnaire for measuring adverse childhood experiences. Furthermore, there were no pronounced sex differences regarding early life stress in the study sample which was unexpected, since females often report a higher prevalence.

There is ongoing debate regarding the optimal measurements to use in cG×E models. On one hand, theories suggest that early life stress impacts individuals throughout their lives. On the other hand, some argue that stressful or traumatic events occurring closer in time have a greater impact in cG×E models (14). In our study, we employed the Neuropattern Questionnaire–Pre-/postnatal-Stress-Questionnaire (NPQ–PSQ) to measure ELS, as reported by parents (56). This measurement tool offers a straightforward and objective evaluation of ELS, which can be challenging to accurately assess through self-reports from adolescents. However, it is important to note that such measures of ELS may not fully capture the individual stress load required to demonstrate effects in a cG×E model.

Furthermore, we employed the parent report of the Parents as Social Context Questionnaire (PASCQ) (54) to measure positive parenting style. This instrument is believed to capture a general parenting style throughout childhood and adolescence, rather than being tied to a specific developmental period. If this assumption holds true, the measure may reflect two aspects: first, the overall parenting approach of the parents, and second, their responsiveness to their child’s varying needs, particularly in response to stressful events.

Indeed, we observed effects of positive parenting style both directly and as cG×E effects in relation to IGT net scores, for both the *5-HTTLPR* and *MAOA* genotypes. These findings support the idea that these genotypes exhibit susceptibility properties in response to environmental influences. Male S-carriers of the *5-HTTLPR* showed lower net scores in the presence of less positive parenting and higher net scores with high positive parenting. Similar patterns were observed for male S-carriers of the *MAOA* genotype. A previous study reported interactions between parental supervision and the *5-HTTLPR* in relation to conceptual flexibility, a measure of executive functioning (32). However, in that study, the L allele demonstrated the strongest effects of parental supervision on conceptual flexibility. The similarities and differences compared to the IGT findings are complex and warrant further exploration.

The lack of significant results related to ELS and the noticeable effects of positive parenting in our study do not diminish the potential importance of ELS or stressful events as significant contributors to decision-making processes. Future studies may reveal significant effects of alternative measures of stress and/or abuse. Furthermore, we acknowledge the ongoing debate regarding risk and protective environmental factors, recognizing that protective factors may simply be observed as risk factors from a different perspective (79). Future research efforts might seek to determine whether positive and negative environmental factors lie on the same continuum or are qualitatively distinct (80), exploring not only the stress-resilience continuum but also considering the dimension of time (14).

The intricate relationship between environmental influences, such as stress and positive parenting, and decision-making highlights the complexity of understanding individual differences in behavioral outcomes. While environmental factors emphasize external contributors, decision-making processes are equally shaped by internal dynamics. One key internal mechanism, particularly relevant to the IGT, is the interplay between emotion and rationality, which serves as a foundation for exploring how these factors guide choices and behaviors. A dominating theory regarding the influence of emotional guidance on the IGT is the somatic marker theory, stating that emotional responses to choice-feedback generates biasing signals (somatic markers) which has the potential to guide decision-making under uncertainty (81). Few studies have considered the relationship between somatic markers and genes on decision-making performance. One study found that almost half of the effect of *5-HTTLPR* on IGT decision-making was mediated by somatic markers (19). Additionally, the somatic marker framework often takes a diathetic approach, implying that defective somatic marker functioning conveys increased susceptibility to maladaptive behaviors, e. g. substance-use disorders or behavioral addictions (82, 83). Based on results from the present study, an intriguing direction for future research would be to further explore the influence of somatic markers on differential susceptibility effects on decision-making.

Our findings revealed a main effect of the *5-HTTLPR*, showing that female S allele carriers had a higher IGT net score under risk. However, no main effect of the *5-HTTLPR* was observed in males. Previous studies have reported mixed findings regarding decision-making impairments among S-allele carriers of the *5-HTTLPR*. Some studies found impairments during the initial 40 trials of the IGT, when participants are exploring the decks without knowing their advantages and disadvantages (17) but also during later trials involving risk-based decisions or in overall IGT scores (5, 84), although findings in the literature are inconsistent (18). In the present study, no main effects of the *MAOA* genotype were found.

The present findings of cG×E effects on IGT performance, combined with previous research, suggest susceptibility properties of the *5-HTTLPR* and *MAOA* genotypes. This indicates that these allelic variants may interact with positive and negative environmental factors, leading to variations in phenotypes depending on environmental influences. Therefore, interpreting main effects of these genotypes should be done cautiously when environmental factors are not considered (37). While the *5-HTTLPR* and *MAOA* are promising candidates, several other loci also influence neural structures associated with reward-related decision-making, including variants regulating dopamine activity. For instance, one study identified an interaction between *5-HTTLPR* and the dopamine receptor D4 gene in relation to decision-making on the IGT (85). Interactions between serotonergic genotypes and variants regulating dopamine, noradrenalin, and glutamate, are also thought to contribute to reward processing and gambling behavior (86, 87). Future research examining the interactions between multiple polymorphisms, rather than individual alleles in isolation, is warranted.

Additionally, factors such as psychiatric diagnoses, neuropsychiatric disabilities, hormonal levels, and other as yet undetermined influences were not controlled for in the present study, and their potential impact warrants further investigation in future research.

The present study has several limitations. Firstly and foremost among these is the issue of sample size, which is a common concern in cG×E studies. The adequacy of sample sizes has been a topic of frequent debate, particularly since the critical review of the first decade of cG×E interaction research by Duncan and Keller (88). Sample sizes typically based on genetic main effects may be appropriate if a clear main effect of a candidate gene on the phenotype of interest exists. However, in studies involving the theories of genetic susceptibility with genes like *5-HTTLPR* and *MAOA*, the relevance of genetic main effects diminishes because they are contingent upon specific environmental factors included in the model, as well as the positive and negative environmental load within the study population (14, 37, 38). Furthermore, others have posited that many well-designed studies testing cG×E hypotheses involve samples of fewer than 300 participants, as smaller samples often allow for improved control over variable assessments and methodological rigor (44, 45). Our study, with its experimental design and objective measurements of the outcome variable, may thereby provide added validity and reliability compared to larger studies relying solely on questionnaire data (44). However, as is often the case, the sample size may have influenced the robustness of the findings and, to some extent, the generalizability of the results. On the other hand, IGT studies very seldom exceeds the sample size of the present study (40). Therefore, the most practical approach to increasing the sample size may involve conducting pooled meta-analyses of future cG×E studies.

Secondly, ELS exhibited severe skewness and included outliers, and neither a log nor a log-log transformation normalized the distribution of the data. Consequently, we opted to dichotomize the ELS variable, which further reduced statistical power but mitigated scaling artifacts. Choosing appropriate statistical methods for cG×E studies is challenging when variables are skewed and measured on ordinal or interval scales. Moreover, since our outcome variable included negative values, approaches like Poisson or negative binomial regression were not suitable. Instead, we employed a General Linear Model with the robust HC0 method for heteroskedasticity-consistent standard errors. Skewed ELS data is a well-documented challenge in cG×E research, where extreme stress levels in a subset of participants can inflate effect sizes or obscure subtle interactions. If the distribution of ELS is indeed skewed, this may have led to an overestimation of the effect of the *MAOA/5HTTLPR* polymorphisms on IGT performance. For instance, participants with low or absent ELS may exhibit baseline IGT performance that is less influenced by genetic variation, potentially confounding comparisons with those who have experienced high levels of ELS. To address these potential biases and ensure robust conclusions in future studies, balanced sampling with respect to ELS experiences, the use of data transformations (e.g., log-transformations), incorporation of additional covariates to account for confounding effects, and the adoption of longitudinal designs would be preferable. However, practical limitations such as the inability to ascertain ELS exposure prior to sampling, constraints on time and funding for large-scale studies, and the logistical challenges of repeated testing over time illustrate the inherent difficulties in conducting such research. Despite these challenges, discovering significant interactions aligning with findings from diverse research fields underscores the importance and implications of our results.

Thirdly, it is challenging to ascertain the representativeness of the participants in the present study. The cohort study, Survey of Adolescent Life in Västmanland (SALVe Cohort), was initiated in 2012, inviting all adolescents born in 1997 and 1999 in Västmanland, Sweden, along with their guardians. The initial response rate was approximately 40% (42, 89). In 2015, wave 2 of data collection was conducted, and participants from this wave were recruited for an experimental session in the present study. To ensure the representativeness of the sample, participants were invited in a randomized order and consecutively included until the final sample size was achieved. Furthermore, the inclusion procedure aimed to achieve an equal distribution of age and birth year among participants.

Fourthly, we utilized the PASCQ and the NPQ–PSQ, both of which are self-reports completed by parents during wave 2 of this cohort study. While parental reports may be subject to bias—since parents might wish to present themselves more favorably and could underreport early life stress events and negative parenting styles—the PASCQ has demonstrated promising validation results (55, 90). In our cohort, the psychometric properties of the parent reports were acceptable, although the association between parent and adolescent reports was weaker, with somewhat better agreement observed among females (55). This suggests that while parental reports may effectively capture the parents’ perspective on the relationship, they may not fully represent the adolescents’ experiences, and vice versa. In this study, we focused on early life stressors, relying on parental reports due to the challenges adolescents face in accurately recalling their exposure to stressors during pregnancy, birth, and early childhood. This approach ensured that both negative and positive life experiences were reported by the same informants. However, other models incorporating adolescent reports on their relationships with parents and more recent negative environmental factors may warrant further investigation regarding their influence on IGT net scores.

Fifthly, our models were not adjusted for alcohol or drug use, current or past mental health issues, or specific diagnoses. One might argue that such adjustments are pertinent in a study of decision-making processes, particularly if these factors influence decision-making abilities. On one hand, if decision-making and these phenotypes share a strong relationship, and if the same cG×E interactions influence both, it raises concerns about the outcome intersection problem (14). On the other hand, if decision-making predicts these phenotypes, adjusting for them could potentially obscure cG×E effects due to stronger statistical associations.

Lastly, in the present study we used a relatively small sample for cG×E in relation to IGT net score, and performed multiple testing given several independent variables of interest. However, due to the exploratory approach, adjusted p-values were not applied. While corrections for multiple testing are critical in confirmatory studies to control the experiment-wise error rate, the resulting reduction in statistical power following such corrections can be counterproductive in exploratory settings and may prevent the detection of true associations (91). There is an ongoing debate regarding the adequacy of power calculations and the usefulness of *post hoc* analyses for multiple comparisons. However, we argue that hypothesis-driven, well-designed studies should focus on interpreting changes in effect sizes due to specific interactions (92), rather than solely relying on power calculations and multiple testing corrections. Additionally, the traditional causal steps approach for interaction tests has low power for detecting intervening effects (93). Hence, the present findings based on crude p-values offer an indication of the susceptibility properties of the *5-HTTLPR* and *MAOA* in IGT performance. To strengthen these conclusions, further investigation through highly powered studies with evenly distributed levels of positive and negative environmental factors is warranted.

Despite its limitations, the present study has several notable strengths. Firstly, it benefits from the robust features of small, well-designed studies, including face-to-face meetings and objective testing of participants, as well as the use of parental reports for variables that adolescents might find difficult to recall. Secondly, the dependent variable, the IGT net score, is a well-studied measure that does not rely on self-reports, thereby minimizing potential biases. Thirdly, the candidate genes investigated in this study, *5-HTTLPR* and *MAOA*, have been extensively researched and have well-established theoretical links to decision-making processes. Fourthly, our findings highlight a clear susceptibility effect, as illustrated in the figures, which may explain the discrepancies in previous research regarding the effects of the L-allele and S-alleles on decision-making outcomes. We posit that results can vary significantly depending on the risk environment of the sample or the presence of multiple environmental resilience factors among participants.

## 5 Conclusion

In this study, we explored the interaction effects of the *5-HTTLPR* and *MAOA* genotypes with positive and negative environmental factors on adolescent decision-making under risk, as measured by the IGT. Our findings indicate that both genes significantly interact with positive parenting, characterized by warmth, structure, and autonomy support, in influencing decision-making processes among males. Specifically, males carrying the S-allele of both genotypes demonstrated notable susceptibility to varying levels of positive parenting, which was reflected in their IGT net scores. These results offer new insights into the role of genetic susceptibility in decision-making and underscore the importance of environmental factors in shaping these processes.

We suggest that future research should continue to investigate the interactions between genetics and both positive and negative environmental factors, rather than seeking main effects of candidate genes in isolation. This approach can help avoid the artifact of environmental variability across different populations, which may lead to conflicting findings in cG×E studies.

In conclusion, our study demonstrates that the *5-HTTLPR* and *MAOA* genes exhibit susceptibility properties in interaction with environmental factors in relation to decision-making processes under risk, as assessed by the IGT. We hope this research paves the way for further advancements in understanding the complex interplay between genetic and environmental factors in shaping human behavior and decision-making.

## Data Availability

The raw data supporting the conclusions of this article will be made available by the authors, without undue reservation.
