# Supplementary material for: Differential susceptibility effects of the 5-HTTLPR and MAOA genotypes on decision making under risk in the Iowa gambling task
Source: Front Psychiatry. 2025 Feb 19;16:1456490. doi: 10.3389/fpsyt.2025.1456490 (PMC11880251; doi:10.3389/fpsyt.2025.1456490)
Supplement: Supplementary file 1 [file Presentation1.pdf]

Supplementary Material

1 Block-wise net-scores in males and females

A repeated measures ANCOVA was performed to explore sex-differences in net-scores across 20-trial blocks of the Iowa gambling task. Model effects showed no significant sex differences in block-wise changes of net-scores across the task. However, post hoc parameter estimates suggested significantly lower net-scores in females compared to males during the final block (trial 81-100) (B = -2.46, CI = -4.80 – -0.11, p = 0.040, η_p^2 = 0.016) (Figure 1).

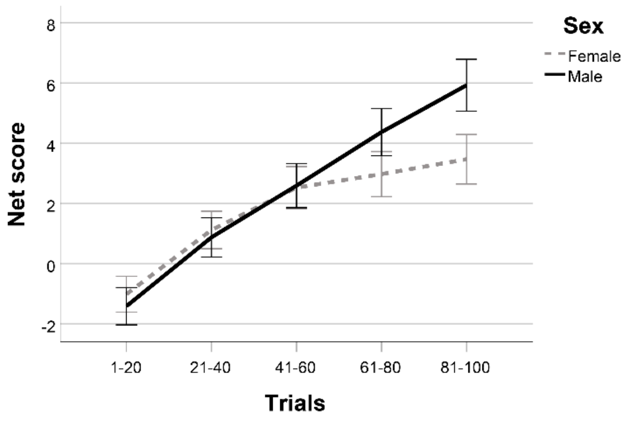

Figure 1. Block-wise net score in males and females. *Error bars:* +/-1 standard error.

2 Influence of genotypes on block-wise net-scores in males and females

Repeated measures ANCOVA was performed in subgroups of males and females to explore the influence of *5-HTTLPR* and *MAOA* genotypes on net-scores across 20-trial blocks of the IGT. Model effects showed no significant differences in block-wise net-scores between *5-HTTLPR* or *MAOA* variants, in males or females (Figure 2 and Figure 3). However, post hoc parameter estimates suggested significantly higher net-scores in female *5-HTTLPR* SS/SL carriers compared to female LL carriers (B =3.13, CI = 0.13 – 6.14, p = 0.041, η_p^2 = 0.032) (Figure 2A).

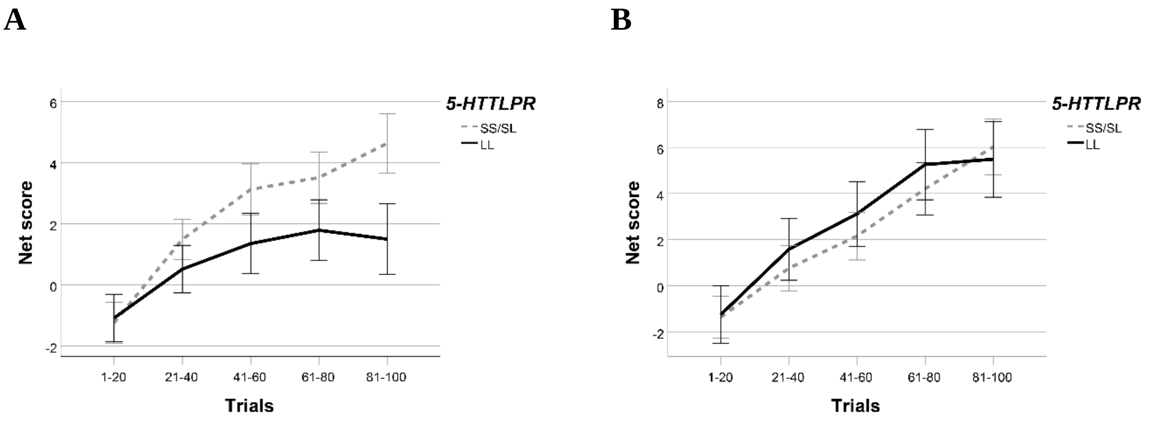

Figure 2. Block-wise net score in *5HTTLPR* variants. A) net-scores in *female* LL and SS/LS. B) net-scores in *male* LL and SS/LS. *Error bars:* +/-1 standard error.

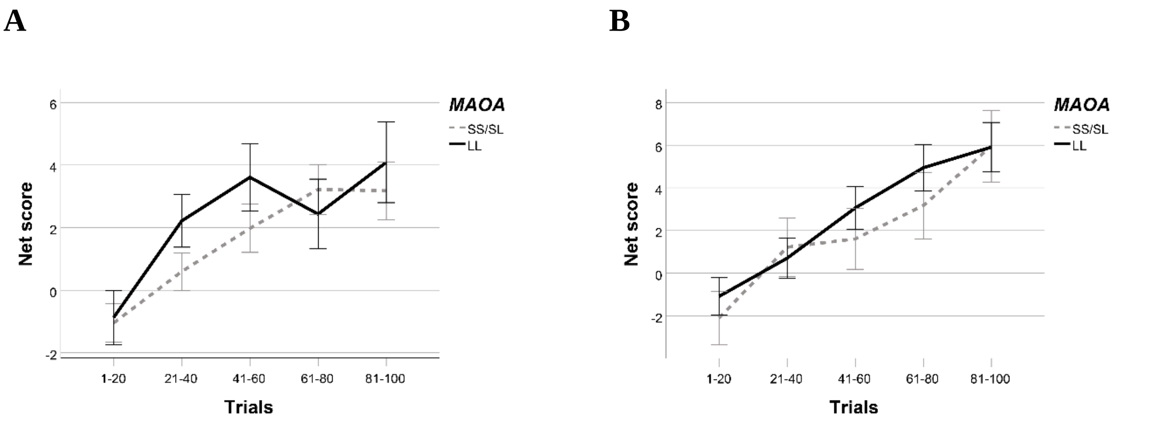

Figure 3. Block wise net score in *MAOA* variants. A) net-scores in *female* LL and SS/LS. B) net-scores in *male* LL and SS/LS. *Error bars:* +/-1 standard error.

3 Genetic analyses

Molecular genetics technology utilises the polymerase chain reaction (PCR) to identify small fragments of DNA that can be manipulated in the laboratory. The process involves the replication of the DNA using of primers – single-stranded DNA molecules complementary to the DNA sequence to be copied. Upon heating, DNA double helix unwinds, and as the mixture cools, the primers bind to the single-stranded genomic DNA through base pairing. This cycle is repeated multiple times, increasing the DNA concentration by 10^5 to 10^6 -fold, enabling the detection of polymorphisms through various genotyping techniques (1).

3.1 *5-HTTLPR*

The *5-HTT* gene is located at approximately chromosome 17q12 (2-5). Transcription of the gene is modulated by the polymorphism *5-HTTLPR,* situated in the upstream promoter region of the *5-HTT* gene. This polymorphism comprises repetitive sequences of varying lengths, containing 20-23-bp repeat elements (6-8). An insertion or deletion event in the *5-HTTLPR* approximately 40 million years ago (9) gave rise to a short (S) 14-repeat allele and a long (L)

16-repeat allele, with the short variant being associated with reduced transcriptional efficiency (6, 7, 10, 11).

3.2 *MAOA-uVNTR*

The MAOA enzyme metabolises serotonin, norepinephrine and dopamine, neurotransmitters that play critical roles in various brain functions, including stress regulation (12, 13). Humans and most other mammals produce two MAO enzymes, MAOA and MAOB (14). These enzymes are encoded by two genes arranged in a tail-to-tail orientation on the X chromosome, specifically between bands Xp11.23 and Xp11.4 (12, 13).

Males possess one X chromosome (inherited from the mother) and one Y chromosome (from the father), whereas females have two X chromosomes (one from each parent). Consequently, males have a single copy of the gene encoding MAOA, while females have two copies. Females may therefore be either homozygous (two similar variants) or heterozygous (two different variants) for the *MAOA* gene.

A variable number of tandem repeats (VNTR) within the *MAOA* gene is located 1.2 kb upstream of the *MAOA* coding sequences. This VNTR consists of a 30 base-pair repeated sequence that can occur in 2, 3, 3.5, 4, or 5 copies (13, 15-17).

3.3 Genotyping procedure

Genomic DNA was extracted from saliva samples using the Oragene Self-Collection Kit (DNA Genotek Inc. Ottawa, Ontario, Canada) with a standard in silica-based method for DNA analyses according to the manufacturer’s guidelines (18, 19).

3.3.1 Genotyping of the *5-HTTLPR*

The *5-HTTLPR* was amplified in a 10 µl reaction mixture containing 30 ng genomic DNA, 1mM PCR Buffer10x with 1.5mM $MgCl_2$, 0.2 µM dNTPs, 0.8 µM of two primers, and 0.5 U FastStart Taq DNA polymerase (Roche Diagnostics GmbH, Mannheim, Germany). The primer sequences were: forward 5´-AAC ATG CTC ATT TAA GAA GTG GAA C-3´ and reverse 5´-XCT AGA GGG ACT GAG CTG GAC AAC -3´. The reverse primer was labelled with the fluorescent dye 5´-hex. PCR reactions were performed on a GeneAmp 9700 (Applied Biosystems Inc., Foster City, California, USA) starting at 94°C for 4 min, followed by 35 cycles of denaturation at 94°C for 45 s, annealing at 61°C for 1 min and elongation at 72°C for 90 s, with a final extension at 72°C for 7 min.

3.3.2 Genotyping of the *MAOA-uVNTR*

The *MAOA-uVNTR* polymorphism was amplified in a 10 µl reaction mixture containing no less than 60 ng DNA, 1x GC-rich buffer, 1x PCR buffer with 2 mM $MgCl_2$, 0.7 mM dNTP, 0.2 µM primers, 0.75 mM $MgCl_2$, and 0.5 U fast start *Taq* DNA polymerase (Roche-Applied Science, Mannheim, Germany).
The primer sequences were: forward 5´-ACA GCC TGA CCG TGG AGA AG-3´ and reverse 5´-GAA CGG ACG CTC CAT TCG GA-3´. The forward primer was labelled with the fluorescent dye 5´-HEX. PCR cycling conditions were hot start for 4 minutes at 95°C followed by denaturation for 30 seconds at 94°C, annealing for 30 seconds at decreasing temperatures from 62 to 55°C with 1°C decrement every 3 cycles and 25 cycles at annealing temperature of 55°C, and extension for 45 seconds at 72°C. Final extension at 72°C for 7 minutes was performed.

3.3.3 PCR analysis

The PCR products were resolved by electrophoresis on 2% agarose in 0.59 TBE buffer, run 2 hours at 110v and visualized using ethidium bromide (SigmaAldrich, St. Louis, MO) under ultraviolet light. PCR product sizes were determined by comparison with 50-bp DNA ladder (VWR, 5 prime GmbH, Hamburg, Germany).

3.4 Classification of genotypes

For the *5-HTTLPR* analysis, individuals were divided into three groups depending on the allelic variation: those homozygous for the short allele (SS), those heterozygous for the short and long allele (LS) and those homozygous for the long allele (LL).

For the *MAOA-uVNTR* analysis, individuals were divided into three groups depending on allelic variation: short alleles with 2 or 3 copies (S/SS), long alleles with 3.5, 4, or 5 copies (L/LL), and the heterozygous female variant of one long and one short allele (LS).

4 References

1. McGuffin P, Owens MJ, Gottesman II. Psychiatric Genetics & Genomics. New York: Oxford University Press; 2002. 492 p.
2. Ramamoorthy S, Bauman AL, Moore KR, Han H, Yang-Feng T, Chang AS, et al. Antidepressant- and cocaine-sensitive human serotonin transporter: Molecular cloning, expression, and chromosomal localization. PNAS. 1993;90:2542-6.
3. Lesch KP, Balling U, Gross J, Strauss K, Wolozin BL, Murphy DL, et al. Organization of the human serotonin transporter gene. Journal of Neural Transmission General Section. 1994;95(2):157-62.
4. Lesch KP, Wolozin BL, Estler HC, Murphy DL, Riederer P. Isolation of a cDNA encoding the human brain serotonin transporter. Journal of Neural Transmission. 1993;91:67-72.
5. Gelernter J, Pakstis AJ, Kidd KK. Linkage mapping of serotonin transporter protein gene SLC6A4 on chromosome 17. Human Genetics. 1995;95:677-80.
6. Lesch KP, Bengel D, Heils A, Sabol SZ, Greenberg BD, Petri S, et al. Association of anxiety-related traits with a polymorphism in the serotonin transporter gene regulatory region. Science. 1996;274(5292):1527-31.
7. Heils A, Teufel A, Petri S, Stober G, Riederer P, Bengel D, et al. Allelic variation of human serotonin transporter gene expression. Journal of Neurochemistry. 1996;66(6):2621-4.
8. Canli T, Lesch K-P. Long story short: the serotonin transporter in emotion regulation and social cognition. Nature Neuroscience. 2007;10(9):1103-9.
9. Lesch KP, Meyer J, Glatz K, Flugge G, Hinney A, Hebebrand J, et al. The 5-HT transporter gene-linked polymorphic region (5-HTTLPR) in evolutionary perspective: alternative biallelic variation in rhesus monkeys. Rapid communication. Journal of Neural Transmission. 1997;104(11-12):1259-66.
10. Lesch KP. Gene-environment interaction and the genetics of depression. Journal of Psychiatry & Neuroscience. 2004;29(3):174-84.
11. Collier DA, Stober G, Li T, Heils A, Catalano M, Di Bella D, et al. A novel functional polymorphism within the promoter of the serotonin transporter gene: possible role in susceptibility to affective disorders. Molecular Psychiatry. 1996;1(6):453-60.
12. Shih JC, Chen K, Ridd MJ. Monoamine oxidase: From genes to behavior. Annual Review of Neuroscience. 1999;22:197-217.
13. Sabol SZ, Hu S, Hamer D. A functional polymorphism in the monoamine oxidase A gene promoter. Human Genetics. 1998;103(3):273-9.
14. Johnston JP. Some observations upon a new inhibitor of monoamine oxidase in brain tissue. Biochemical Pharmacology. 1968;17(7):1285-97.
15. Deckert J, Catalano M, Syagailo YV, Bosi M, Okladnova O, Di Bella D, et al. Excess of high activity monoamine oxidase A gene promoter alleles in female patients with panic disorder. Human Molecular Genetics. 1999;8(4):621-4.
16. Denney RM, Koch H, Craig IW. Association between monoamine oxidase A activity in human male skin fibroblasts and genotype of the MAOA promoter-associated variable number tandem repeat. Human Genetics. 1999;105(6):542-51.
17. Huang YY, Cate SP, Battistuzzi C, Oquendo MA, Brent D, Mann JJ. An association between a functional polymorphism in the monoamine oxidase a gene promoter, impulsive traits and early abuse experiences. Neuropsychopharmacology. 2004;29(8):1498-505.
18. Bendre M, Comasco E, Checknita D, Tiihonen J, Hodgins S, Nilsson KW. Associations between MAOA-uVNTR genotype, maltreatment, MAOA methylation, and

alcohol consumption in young adult males. Alcoholism: clinical and experimental research. 2018;42(3):508-19.
19. Åslund C, Leppert J, Comasco E, Nordquist N, Oreland L, Nilsson KW. Impact of the interaction between the 5HTTLPR polymorphism and maltreatment on adolescent depression. A population based study. Behavior Genetics. 2009;39:524-31.
